# Effects and Mechanisms Investigation of Heat Stress on Egg Yolk Quality in Huaixiang Chickens

**DOI:** 10.3390/ani13223513

**Published:** 2023-11-14

**Authors:** Yuxia Chen, Sumeng Yu, Li Zhang, Mei Xiao, Lilong An

**Affiliations:** Department of Animal Science, College of Coastal Agricultural Sciences, Guangdong Ocean University, Zhanjiang 524088, China; chenyuxia@gdou.edu.cn (Y.C.); ysmlx123@163.com (S.Y.); zhangli761101@163.com (L.Z.); xiao0812@126.com (M.X.)

**Keywords:** heat stress, egg yolk quality, lipid metabolism, Huaixiang chickens

## Abstract

**Simple Summary:**

High ambient temperature is a significant factor contributing to economic losses in the poultry industry. Heat stress had adverse effects on the growth performance, reproductive performance, and egg production of poultry birds. However, the specific impact of heat stress on internal egg yolk quality parameters and the potential mechanisms of local chickens remain to be further studied. The purpose of this study was to explore the influences of heat exposure on internal egg yolk quality parameters and their possible mechanisms in Guangdong Province Xinyi Huaixiang chickens. Our results revealed that heat exposure (32 °C) for 6 weeks reduced yolk weight, yolk color, and egg weight. Additionally, we observed an upregulation of lipid metabolism-related genes, including SREBP-1c, ACACA, and FASN. These results provided valuable insights into the underlying mechanisms of egg yolk quality changes induced by heat stress. Understanding these mechanisms is crucial for mitigating the negative impact of heat stress on poultry production and improving the overall performance and well-being of poultry birds.

**Abstract:**

The purpose of this study was to examine the effects of high temperature on internal egg yolk quality parameters and their possible mechanisms in Huaixiang chickens. This study consisted of two treatments, and each treatment had six replicates with six birds per cage. A total of seventy-two 26-week-old female Huaixiang chickens were randomly divided into a normal-temperature group (NT) and a high-temperature group (HT) for 6 weeks. And these hens were exposed to 25 ± 2 °C and 32 ± 2 °C, respectively. Their relative humidity was maintained at 55–65%. The results showed that the HT group significantly reduced yolk weight, yolk color, and egg weight compared to the NT group (*p* < 0.05). Heat stress caused vacuolar degeneration of the liver and reduced the absolute liver weight (*p* < 0.05). Both yolk triglyceride (TG) and liver TG in the HT group were significantly higher than in the NT group (*p* < 0.05). However, the liver total cholesterol (TC) level in the HT group was remarkably lower than that in the NT group (*p* < 0.05). Additionally, heat stress remarkably enhanced SREBP-1c, ACACA, and FASN lipid metabolism-related gene mRNA expression levels in Huaixiang chicken liver after 6 weeks of heat exposure (*p* < 0.05). Furthermore, the HT group had remarkably reduced total amino acid, Cys, and Tyr levels in the yolk when compared with the NT group in our experiment (*p* < 0.05). In conclusion, heat stress causes egg yolk quality reduction and abnormal lipid metabolism in Huaixiang chickens. These findings provided novel insights into the role of high temperature on egg yolk parameters and the underlying mechanisms in Chinese indigenous laying hens.

## 1. Introduction

Heat stress is the most significant environmental challenge for the modern poultry industry worldwide. Among monogastric animals, birds are particularly susceptible to high temperatures compared to other monogastric animals, which is primarily due to their feather coverage and absence of sweat glands [1]. Kocaman and Balnave et al. reported that the optimal temperature range for laying hens is typically between 16 and 25 °C [2,3]. Heat stress could occur when birds adapted to lower ambient temperatures encounter rising temperatures above 25 °C. This phenomenon leads to detrimental effects on the physiological and performance traits of poultry [4]. The total annual economic loss caused by heat stress in the United States poultry industry was estimated to be between USD 128 and 165 million [5]. Additionally, in many countries, poultry routinely experience temperatures reaching 32 °C and higher. Especially in the southern regions of China, where summers are long, the high temperatures disrupt the thermoregulation process in poultry [6]. Xinyi Huaixiang chicken, a special breed found in the western part of Guangdong Province, southern China, possesses commercial advantages such as huge market demand, extensive feeding, and a huge development prospect. It serves as the favorite chicken breed of local farmers in Guangdong and contributes significantly to egg production [7].

Overall egg quality is important for both poultry breeders and consumers in the global egg industry. When assessing egg quality, two main aspects are taken into consideration: external and internal traits [8,9]. External characteristics, such as egg weight and the condition of the shell, play a critical role in determining consumers’ acceptability [10]. Simultaneously, the interior parameters of eggs, specifically yolk index, including yolk weight, yolk color, yolk amino acid, and so on, hold significant significance as good nutrition criteria in the egg production industry.

In recent decades, climate change has notably contributed to an increase in the occurrence of hot days. It has been generally thought that high temperatures have detrimental effects on poultry egg production. Many studies have mainly focused on examining the effects of heat stress on the egg’s external qualities, while studies investigating the effects of heat stress on internal egg quality and the underlying mechanisms of local chickens under heat exposure are limited in the present literature. Therefore, this research aimed to investigate the impact of high temperature on the internal egg yolk quality traits, mainly including yolk weight, yolk color, yolk lipids, and yolk amino acid, and the potential mechanisms of Huaixiang chickens.

## 2. Materials and Methods

### 2.1. Animals, Housing, Experiment Design, and Diet

Seventy-two 26-week-old Chinese indigenous young yellow-feather laying hens (Huaixiang chickens) were bought from the Guangzhou poultry industry in China. The experiment was terminated when they were 33 weeks old (weight of 1717 g). Chickens at 26 weeks of age were randomly assigned to 2 experimental groups with six replicates of six birds per replicate. And these chickens were divided into two groups, including the normal-temperature group (NT) and the high-temperature group (HT), and they were fed a basal diet (Table 1). The pre-trial period was 2 weeks (26–27 weeks), and the trial period was 6 weeks (28–33 weeks). All the birds were kept at a temperature of 25 ± 2 °C with 55–65% relative humidity during the pre-trial period. In the trial period, the NT group was maintained at a temperature of 25 ± 2°C with 55–65% relative humidity. The hens in the HT group were maintained under controlled conditions, with a temperature of 32 ± 2 °C and a relative humidity of 55–65% for 6 weeks from 10:00 a.m. to 6:00 p.m. for 8 h each day, and they were kept at 25 ± 2 °C for the rest of the day. The chickens were kept in cages with soft bedding and housed in a well-ventilated room that had been disinfected with potassium permanganate and formalin. The temperature and relative humidity at 5 positions in the chicken house were measured 3 times per day. Artificial light was kept for 16 h a day (from 7:00 to 23:00) at 10–15 Lx during the whole experiment. We installed a conditioner, incandescent lamps, and a dehumidifier in every chicken house in order to control environmental parameters based on the chickens’ physiological needs with no limitations on feed or water.

At 33 weeks, on the last day of this experiment, all birds were fasted overnight, and body weight was measured. The hens were sacrificed by overdose anesthetic injection, the liver tissues were removed and weighed, and samples were either snap frozen in liquid nitrogen or fixed with 4% paraformaldehyde for later analysis.

### 2.2. Sample Collection and Storage

One egg was randomly selected from each replicate at 6:00 p.m. on Sunday from each group of the 6th week. And the surface of the selected egg was intact and clean; after being labeled, they were placed in a 4 °C refrigerator for temporary storage.

After gently cracking the egg, the egg white was carefully filtered, and the egg yolk was then gently placed into a clean and dry glass Petri dish. We carefully absorbed the excess egg white near the egg yolk with a needle tube to obtain a complete egg yolk, which was used for the determination of subsequent test indicators.

### 2.3. Egg Yolk Quality Indicators Measurement

The selected eggs and the separated yolk were weighed using an analytical balance, and the measurements were recorded at room temperature. The ratio of yolk weight to egg weight was also calculated by recording data. The egg yolk color was measured based on the DSM Yolk Color Fan (DSM Nutritional Products, Basel, Switzerland), which ranges from pale yellow at score 1 to dark orange at score 15 [12].

### 2.4. Yolk and Liver Lipids Analysis

The yolk and liver total cholesterol (TC), triglyceride (TG), and HDL cholesterol (HDL-C) contents were detected via enzymatic colorimetric methods of commercial kits (Grace Biotechnology, Suzhou, China). LDL cholesterol (LDL-C) was determined according to the formula of Friedewald et al. [13]. The TG and TC in the liver were quantified with 10% liver homogenate.

### 2.5. Histological Examination

Liver tissue morphology analysis was carried out using hematoxylin–eosin (HE) staining [14]. Briefly, liver tissues were fixed in 4% paraformaldehyde (PFA) for 12 h and subsequently embedded in paraffin. Then, they were sliced into 4 μm thick sections using a Leica microtome (Nussloch Gmbh, Nußloch, Germany). These sections were dehydrated using various contents of ethanol and xylol, briefly washing. To stain the cell nuclei, 5% hematoxylin was applied for 10 min. After a 5 min rinse in distilled water, the sections were incubated in 0.1% HCl–ethanol for 30 s. The tissues were then stained for 2 min. After washing and dehydration, the stained tissue sections were checked using light microscopy (Olympus BX51, Tokyo, Japan) equipped with a digital camera (DP72; Olympus). The remaining fresh liver tissues were frozen and then stored at −80 °C for qRT-PCR analysis.

### 2.6. Quantitative Real-Time PCR Analysis

The total RNA of liver tissues was isolated based on the protocol instructions. Then, it was treated with RNase-free DNase and reversely transcribed through the first-strand cDNA Synthesis Kit (TransStart; transgen.com.cn (accessed on 1 October 2023)). In brief, 0.4 μL RNA was combined with 1 μL of reverse transcriptase primers (0.2 μg/μL), 2 μL of dNTP Mix (10 mmol/L), 4 μL of 5× Reaction Buffer, 1 μL of RT (200 U/μL), 1 μL of RNase Inhibitor (20 U/μL), and 10.6 μL of RNase free ddH2O. The final reaction mixture was heated to 42 °C for 50 s, then kept at 85 °C for 15 min, and ultimately cooled to 4 °C. To perform quantitative analysis of specific gene mRNA expression from the liver tissue samples, the cDNA was subjected to qPCR using the LightCycle instrument (Roche, Basel, Switzerland). The reaction mixture (20 μL) included 1 μL of cDNA sample, 0.4 μL of forward and reverse primer, 10 μL of SYBR Green PCR master mix, and 8.2 μL of nuclease-free water. The PCR products were detected via 1% agarose gel to ensure gene sequencing and assess their specific temperature requirements. The qRT-PCR experiments were carried out using a Bio-Rad CFX Connect real-time PCR detection system (Bio-Rad, Hercules, CA, USA). The following amplification conditions were used for qRT-PCR: pre-denaturation at 94 °C for 30 s, 40 cycles of denaturation at 94 °C for 5 s, annealing at 55 °C for 15 s, and elongation at 72 °C for 10 s. The primer sequences used for sterol regulatory element-binding proteins (SREBP), Ace-CoA Carboxylase (ACACA), fatty acid synthase (FASN), liver X receptor α (LXRα), and malic enzyme (ME) in these assays are listed in Table 2. They were designed using Primer Premier 5.0 and synthesized by Shanghai Sangon Biotech Co., Ltd. (Shanghai, China) [15]. The mRNA levels were calculated based on the blank control after normalizing to the β-actin using the 2^−ΔΔCT^ approach [16]. All analyses were performed in triplicates.

### 2.7. Yolk Amino Acid Traits

Yolk solution (7 g) was placed in a 10 mL centrifuge tube and sent to Grace Biotechnology Co., Ltd. for testing (Suzhou, China). The total amino acids were detected via the enzymatic colorimetric methods of commercial kits (Grace Biotechnology, Suzhou, China). Amino acids were measured via HPLC (LC-100; Shanghai Wufeng Co., Ltd., Shanghai, China). An amino acid standards solution (types H and B), L-aspartic acid, and L-glutamic acid were prepared according to the same protocol. Before analyzing every 30 samples, standard samples were analyzed as a quality control measure. The amino acids content in the yolk was determined by calculating the peak ratio between the sample and the respective standard.

### 2.8. Statistical Analysis

All data were analyzed via one-way analysis of variance (ANOVA) and Duncan’s multiple comparisons post hoc tests (Duncan, 1955) [17]. The Statistical Package for Social Science (SPSS) software version 22.0 was utilized for the statistical analysis. The Kolmogorov–Smirnov test was used for normal distribution analysis. The statistical differences were considered significant if *p* < 0.05 and were regarded as a trend when the *p*-value was in range of 0.05–0.10.

## 3. Results

### 3.1. Effects of High Temperature on Egg Weight-Related Indexes of Huaixiang Chicken

The results demonstrated that the yolk weight was notably reduced by 14.37% in the HT group compared with the NT group (*p* < 0.05). In addition, the egg weight of the HT group significantly decreased by 11.57% in the 6 weeks of the experiment compared with the NT group (*p* < 0.05). However, the HT group had no obvious difference on the yolk weight/egg weight ratio compared with the NT group during the entire testing period (Table 3).

### 3.2. Effects of High Temperature on Yolk Color of Huaixiang Chicken

**Figure 1:**
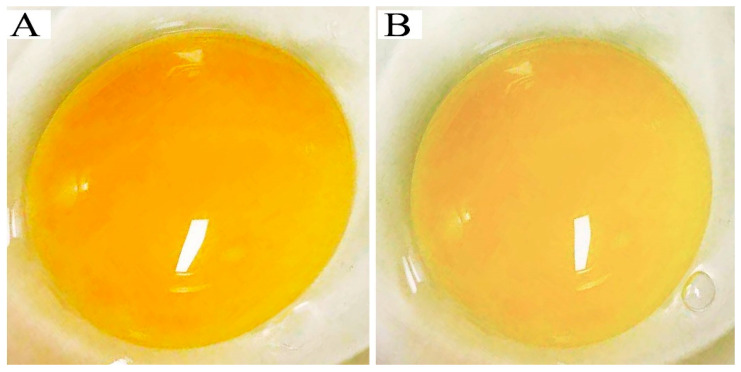
Effect of heat stress on the yolk color in Huaixiang chicken. (**A**) Normal-temperature group (NT); (**B**) high-temperature group (HT).

In our study, the results indicated that the yolk color in the HT group significantly reduced by 18.69% compared to the NT group during this experiment (*p* < 0.05) (Figure 1, Table 4).

### 3.3. Effects of High Temperature on Yolk Lipids of Huaixiang Chicken

The current study found that the TG level in the HT group was remarkably elevated by 27.96% compared to the NT group in the 6 weeks (*p* < 0.05). However, there were no obvious differences observed in the TC, LDL-C, and HDL-C concentrations between the HT group and NT group during the entire experiment (Table 5). These results indicated that heat stress could significantly enhance lipid deposits in yolk of Huaixiang chicken.

### 3.4. Underlying Mechanisms of High Temperature on Yolk Lipids Changes of Huaixiang Chicken

The liver is acknowledged as a crucial organ responsible for regulating lipid metabolism. In our study, we hypothesized that the impact of high temperatures on the yolk lipids changes of Huaixiang chickens might be affected by the regulation of lipid metabolism in the liver. To investigate this hypothesis, we performed the following experiments.

#### 3.4.1. Effects of Heat Temperature on the Liver Histological Changes of Huaixiang Chicken

**Figure 2:**
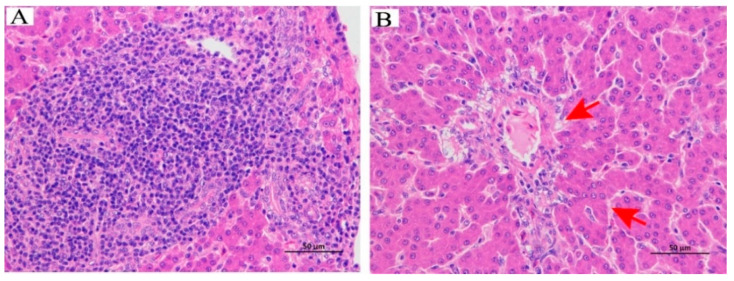
Effect of high temperature on the histopathological changes of liver in Huaixiang chicken (HE staining; scale bar: 50 μm). (**A**) Normal-temperature group (NT); (**B**) high-temperature group (HT). The red triangle represents vacuolar degeneration of the hepatocyte. *n* = 6 for each group.

HE staining was conducted to show histopathological changes of the liver, and the results are presented in Figure 2. Following 6 weeks of heat exposure, the HT group exhibited a marked vacuolar degeneration of hepatocytes, while no significant pathological changes appeared in the NT group. These findings indicated that heat stress significantly contributes to liver tissue damage.

#### 3.4.2. Effects of High Temperature on the Liver Lipids of Huaixiang Chicken

**Figure 3:**
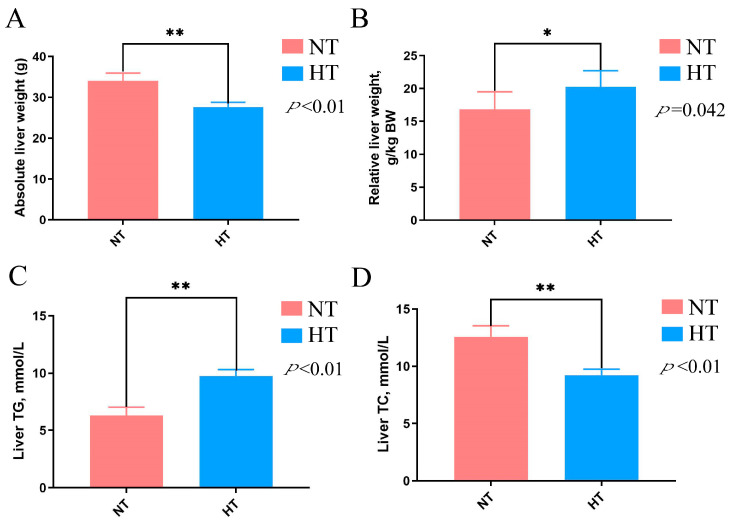
Effects of high temperature on hepatic lipid metabolism of Huaixiang chicken. (**A**) Absolute liver weight; (**B**) relative liver weight; (**C**) TG, triglycerides; (**D**) TC, total cholesterol; normal-temperature group (NT); high-temperature group (HT). Data are the means ± SEM (*n* = 6). * *p* < 0.05 and ** *p* < 0.01.

The results of this study indicated that the HT group had significantly reduced absolute liver weight compared with the NT group during the entire experiment period (*p* < 0.01) (Figure 3A). However, when considering the relative liver weight, the HT group had a higher value compared to the NT group (*p* < 0.05) (Figure 3B). Furthermore, the measurement of the liver TG concentrations showed that the HT group had higher levels than the NT group during the whole experiment (*p* < 0.01) (Figure 3C). However, after 6 weeks of HT treatment, the TC levels in the HT group obviously reduced compared with the NT group (*p* < 0.01) (Figure 3D). These results demonstrated that high temperature remarkably influenced hepatic lipid metabolism.

#### 3.4.3. Effects of High Temperature on Lipid Metabolism Gene Expressions in the Liver of Huaixiang Chicken

**Figure 4:**
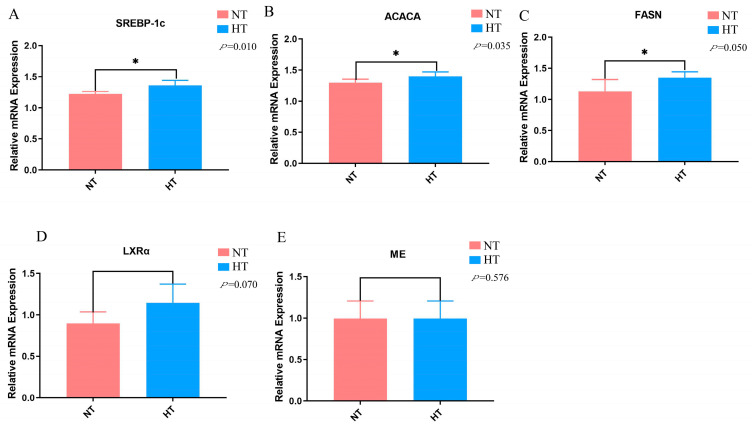
Effect of heat temperature on liver lipid gene expressions. (**A**) SREBP-1c, sterol regulatory element-binding protein 1c; (**B**) ACACA, Ace-CoA Carboxylase; (**C**) FASN, fatty acid synthase; (**D**) LXRα, liver X receptor; (**E**) ME, malic enzyme. Data are the means ± SEM (*n* = 6 for each group). * *p* < 0.05.

The mRNA expression levels of the liver lipid metabolism-associated genes are depicted in Figure 4. After 6 weeks of heat exposure, the results revealed that the HT group increased the mRNA levels of SREBP-1c, ACACA, and FASN in the liver of Huaixiang chickens compared to the NT group (*p* < 0.05). Additionally, LXRα mRNA expression levels showed an increased trend under heat stress (*p* = 0.070). However, no obvious differences were observed in the gene levels of ME in the liver of Huaixiang chickens when comparing the HT group to the NT group. These results further revealed that high temperature remarkably affected hepatic lipid metabolism.

### 3.5. Effects of High Temperature on Yolk Amino Acid of Huaixiang Chicken

Seventeen kinds of amino acids and total amino acid were analyzed in the egg yolk samples: lysine (Lys), phenylalanine (Phe), methionine (Met), threonine (Thr), isoleucine (Ile), leucine (Leu), valine (Val), alanine (Ala), arginine (Arg), glycine (Gly), glutamic acid (Glu), cysteine (Cys), tyrosine (Tyr), proline (Pro), serine (Ser), aspartic acid (Asp), and histidine (His) (Table 6). The results of the present study indicated that the HT group had total amino acid concentrations reduced by 14.32% compared with the NT group during our experiment (*p* < 0.05). Furthermore, Cys and Tyr levels in the HT group had also significantly decreased by 58.73% and 10.03%, respectively, compared with the NT group in our experiment (*p* < 0.05). However, there were no significant differences observed in the other 15 kinds of amino acids between the HT group and the NT group during the whole experimental period.

## 4. Discussion

This experiment was primarily performed to study the impact of high ambient temperature on the internal egg yolk quality and the potential mechanisms in Huaixiang chicken. The intensity and duration of heat exposure play a crucial role in determining the detrimental effects on these growth parameters [18]. Previous studies have indicated that heat stress reduced feed intake and affected the digestibility of different diets [19]. In the current study, our results showed that eggs from the NT group were heavier than those of the high-ambient-temperature group. These results were similar to Star, who reported that heat stress significantly decreased egg weight compared with the hens in the control chamber [20]. It is also consistent with other reports that have demonstrated that heat stress decreased egg weight in commercial laying hens [21,22]. However, these findings are in contrast to the previous results by Barrett et al., which found that the heat-stressed (HS) group had increased egg weight compared to the pre-heat-stressed (pre-HS) group in laying hens [23]. These differences in results may be attributed to heat stress causing feed intake reduction in chickens, limiting the accumulation of egg nutrients and subsequently reducing egg weight.

Egg yolk color is a crucial visual feature that significantly influences consumer perception and has an essential role in the marketing and quality evaluation of the eggs. Apart from enhancing aesthetic appeal, egg yolk color serves as an indicator of an egg’s nutritional composition. Previous studies showed that yolks with deeper hues generally contain higher levels of essential nutrients, such as vitamins A, D, E, and omega-3 fatty acids [12,24]. Additionally, the uniform yellow color of the yolk signifies the health of the chicken flock on the farm. The yolk color primarily depends on the diet of the chickens, specifically the type and amount of consumed xanthophyll pigments [25]. In our study, we found a significant reduction in yolk color in the high-temperature group (HT) compared with the normal-temperature group (NT) of Huaixiang chickens in the experiment. This finding differs from the study conducted by Gholizadeh, who reported that heat stress had no significant impact on egg color with a basal diet [23]. One possible explanation for our research findings is that heat stress reduced feed intake and digestive capacity, resulting in decreased pigment intake and ultimately leading to a reduction in yolk color. Furthermore, heat stress may have damaged liver function, consequently decreasing the synthesis of β-carotene and egg yolk pigmentation. Our study also revealed that high-temperature treatment remarkably elevated the yolk triglyceride (TG) level of Huaixiang chicken. Meanwhile, limited studies showed the mechanisms of heat exposure on the yolk lipid profile of laying hens in the literature. Our results indicated that heat stress significantly enhanced lipid deposits in the yolk of Huaixiang chicken. The phenomenon might be due to adipose tissue lacking fat mobilization, while its ability to respond to lipolysis stimuli was reduced [26].

In order to further explore the underlying mechanisms of heat stress on internal egg yolk quality, we investigated the effect of heat stress on the liver function and hepatic lipid metabolism of Huaixiang chickens. The liver is regarded as an organ that plays a significant role in avian growth and thermoregulation, making it a compelling subject of study due to its high susceptibility to heat stress. Organ weight can serve as an indicator of organ development, thereby reflecting its functionality. In our study, we observed that heat stress remarkably reduced the absolute and relative liver weight in Huaixiang chickens. The results indicated that high temperature detrimentally affects liver growth and development in the local chickens. These findings are consistent with those of Chen et al. who reported that heat stress reduced liver weight in broilers when compared to a control group [27]. Furthermore, Liu et al. demonstrated that heat exposure for two weeks reduced the liver weight and liver index of the broilers [28]. The essential cause behind the harmful influence of high temperature is the reduction in feed intake and nutrients available for the organ growth. In contrast, Ma et al. demonstrated a significant increase in the relative liver weight following heat exposure in broiler chickens when compared to the normal-temperature group [29]. This abnormal increase in liver weight could be attributed to compensatory hypertrophy in response to heat stress to make up for the worse liver function caused by heat exposure.

In poultry, the liver plays a pivotal role as the primary organ for fat synthesis. To gain insights into the impact of heat stress, we conducted histological analysis of the liver using hematoxylin and eosin (HE) staining. Our results unveiled significant vacuolar degeneration within hepatocytes in the HT group of Huaixiang chickens. Similar to our findings, Liu et al. observed that heat stress induced liver tissue damage, including the infiltration of lymphocytes and neutrophils in broilers [28]. Given the liver’s crucial role in digestion and nutrient absorption, such hepatic damage has the potential to impede these vital processes in laying hens. Furthermore, our study revealed that heat stress led to a remarkable increase in the TG levels in the liver, signaling abnormal hepatic fat deposition in chickens. Intriguingly, after six weeks of heat exposure, we noted a substantial reduction in total cholesterol (TC) levels in the HT group’s livers compared to the non-temperature-treated (NT) group. These parallel changes in lipid profiles, characterized by elevated TG levels and decreased TC levels in both the yolk and liver, suggest a potential interconnected and coordinated regulation of lipid metabolism between these two vital organs. In contrast, Yin et al. reported an opposing trend, as they observed a significant increase in TC levels in heat-stressed birds, which is accompanied by a simultaneous decrease in TG levels when compared to birds maintained under normal conditions [30]. The potential reasons for the elevation of hepatic TG levels and the decrease in TC levels induced by heat stress can be analyzed as follows. Firstly, during heat stress, the body requires more energy to cope with the stress response and maintain normal functions. To meet this demand, the liver increases fat breakdown, leading to an elevation in triglyceride levels. Additionally, heat stress leads to the depletion of glycogen, the storage form of glucose, as it is consumed to meet energy needs. In response, the liver utilizes fatty acids for energy production, leading to enhanced triglyceride synthesis and accumulation. Furthermore, heat stress can induce alterations in the levels of hormones and cytokines, which may in turn suppress cholesterol synthesis in the liver. These observations provide a plausible explanation for how heat stress can lead to metabolic disorders, potentially stemming from excessive fat deposition during the experiment.

Some previous studies have reported that heat stress may promote fat synthesis and deposition in broilers [31,32]. Further investigating the underlying mechanisms requires establishing a precise understanding of the interplay between lipid metabolism in the yolk and liver, which contributes to a comprehensive understanding of the physiological responses in avian species during heat stress. Our study analyzed the levels of lipid-related genes in the liver. Our results demonstrated that heat stress significantly increased the mRNA levels of sterol regulatory element-binding protein 1c (SREBP-1c), Ace-CoA carboxylase (ACACA), and fatty acid synthase (FASN) when compared to the NT group in the livers of the Huaixiang chickens. Notably, the mRNA expression levels of live X receptor alpha (LXRα) showed an increasing trend under heat stress conditions. The increased expression of SREBP-1c correlates with increased TG levels in the liver [28]. Both LXRα and SREBP-1c can further activate the transcription of the key genes associated with fat synthesis, including FASN and ACACA [33,34]. ACACA plays a pivotal role in catalyzing the synthesis of malonyl-CoA, while FASN determines the speed of fatty acid synthesis [35]. Similarly, Lu et al. have demonstrated that heat exposure markedly enhanced the mRNA levels of SREBP-1c, ACACA, and FASN in the liver [36]. Consequently, it is plausible that heat stress activates the lipid signaling pathway in the liver, ultimately leading to yolk fat synthesis in Huaixiang chickens. These findings collectively shed light on the potential mechanisms underlying heat stress-induced lipid changes in egg yolks.

The effect of heat stress on amino acids in egg yolk has not been reported until now. To further explore the underlying mechanisms of heat stress on internal egg yolk quality, we investigated the effect of heat stress on yolk amino acids (AAs) of Huaixiang chickens. The concentration of free amino acids primarily hinges on the balance between the formation and degradation of amino acids in the tissues. In our study, we observed a significant reduction in the total amino acid levels in the yolk of the HT group when compared to the NT group. The pathophysiological changes induced by heat stress may increase the amino acid energy supplement requirement and heat shock protein synthesis of laying hens, which leads to the decrease in AA concentration in egg yolk under heat stress conditions [37]. Additionally, we found that the cysteine (Cys) and tyrosine (Tyr) levels were obviously higher in the HT group than in the NT group during our experiment. Cys is recognized as a potential marker and a critical intracellular antioxidant for heat stress. However, research on the specific role of cysteine under heat stress conditions remains limited. Consequently, further studies are imperative to elucidate its functions in heat-stressed animals. Cervantes et al. reported that the serum Cys concentration decreased in heat-stressed growing pigs [38]. The decline in cysteine concentration may be associated with its role as a component of glutathione, a crucial antioxidant compound, along with glutamate (Glu) and glycine (Gly). Cysteine plays a pivotal role in scavenging and neutralizing reactive oxygen species (ROS). ROS production escalates within cells under heat stress conditions, resulting in a higher demand for antioxidants [39]. Cys is a pivotal amino acid that can be catabolized through various pathways, including gluconeogenesis, to produce energy. Moreover, Cervantes et al. also demonstrated that heat stress significantly reduced the essential AA Tyr level in the serum of growing pigs. These results suggest that cells may remove these essential AAs to generate body proteins to relieve the effects of heat stress [38]. We speculate that Cyr and Tyr level changes in egg yolk may be associated with reduced feed intake and nutrient absorption, which are common phenomena in HS birds [40,41]. The increased demand for energy during heat stress can trigger enhanced amino acid catabolism, including Cys and Tyr, consequently resulting in a reduced concentration of these amino acids in the egg yolk. Further research is needed to explore strategies for enhancing the availability of essential amino acid in heat-stressed birds. Such efforts could ultimately improve egg yolk quality and the overall health of chickens.

## 5. Conclusions

The findings of this study indicated that heat stress has a notable adverse impact on the internal quality of egg yolks, especially in the metabolism of yolk lipids deposition in Huaixiang chickens. When compared to the control group, the high-temperature group significantly reduced egg weight, yolk weight, and yolk color as well as the levels of total amino acids, especially Cys, and Tyr in the yolk. The liver plays a critical role in the regulation of lipid metabolism throughout the body, and changes in the liver’s lipid metabolism can potentially influence the lipid profile in the yolk. Notably, there was a similar trend in the expression of triglycerides in both the liver and yolk. Furthermore, heat stress remarkably enhanced the mRNA expression levels of lipid metabolism-related genes, including SREBP-1c, ACACA, and FASN, in the liver of Huaixiang chicken. These gene expression changes partly account for the observed alterations in lipid content in both organs. In summary, these results provided new perspectives for the study of the effects of high temperatures on egg yolk characteristics and the underlying mechanisms in Chinese indigenous laying hens, and they provide a theoretical reference for local laying hens to explore reasonable and effective ways to cope with heat stress.

## Figures and Tables

**Table 1:** Ingredients and chemical composition of the experimental basal diet (air-dry basis).

Ingredients	Percent (%)	Nutrients ^2^ (Analyzed Composition, %)	Content
Corn	55.0	ME (MJ/kg)	11.3
Soybean meal	20.0	CP, %	15.5
Wheat bran	9.5	Ca, %	3.0
Fish meal	5.0	TP, %	0.6
Limestone	7.5	Met, %	0.4
Ca(HCO_3_) ^2^	2.5	Cys, %	0.3
NaCl	0.1	Lys, %	0.8
Premix ^1^	0.4		

Notes: ^1^ The premix provided the following per kilogram of diet: vitamin A 9000 IU, VD 2500 IU, VE 20 IU, vitamin B 12 12 μg, VK 2.4 mg; Microelements: Mn 100 mg, Zn 60 mg, Fe 25 mg, Cu 5 mg, Co 0.1 mg (sulfates form); Se(N_2_SeO_3_·5H_2_O) 0.2 mg, I (KI) 0.5 mg. ^2^ Calculated values were in accordance with the NRC (1994) [11] values for feedstuffs.

**Table 2:** Primer sequences used for real-time PCR.

Genes ^1^	Primer Sequence (5′-3′)	Product Size (bp)
SREBP-1c	F: GCCCTCTGTGCCTTTGTCTTC	130
	R: ACTCAGCCATGATGCTTCTTCC	
ACACA	F: AATGGCAGCTTTGGAGGTGT	136
	R: TCTGTTTGGGTGGGAGGTG	
FASN	F: CGCAGTTTGTTGATGGTGAG	179
	R: TCCTTGGTGTTCGTGACG	
LXRα	F: GTCCCTGACCCTAATAACCGC	186
	R: GTCTCCAACAACATCACCTCTATG	
ME	F: TGCCAGCATTACGGTTTAGC	175
	R: CCATTCCATAACAGCCAAGGTC	
β-actin	F:CAACACAGTGCTGTCTGGTGGTAC	199
	R: CTCCTGCTTGCTGATCCACATCTG	

^1^ SREBP-1c, sterol regulatory element-binding protein 1c; ACACA, Ace-CoA carboxylase; FASN, fatty acid synthase; LXRα, liver X receptor α; ME, malic enzyme.

**Table 3:** Effects of high temperature on egg weight-related indexes of Huaixiang chicken.

Items	NT	HT	SEM	*p*-Value
Egg weight (g)	44.33	39.20	1.00	0.003
Yolk weight (g)	15.94	13.65	0.50	0.012
Yolk weight/egg weight (%)	0.36	0.35	0.01	0.483

NT: normal-temperature group; HT: high-temperature group. *p* < 0.05 was considered to be statistically significant. *n* = 6 for all groups.

**Table 4:** Effects of high temperature on yolk color of Huaixiang chicken.

Items	NT	HT	SEM	*p*-Value
Yolk color	7.17	5.83	0.289	0.012

NT: normal-temperature group; HT: high-temperature group. *p* < 0.05 was considered to be statistically significant. *n* = 6 for all groups.

**Table 5:** Effects of high temperature on yolk lipids of Huaixiang chicken.

Items	NT	HT	SEM	*p*-Value
TG mg/g	29.43	37.66	2.06	0.016
TC mg/g	10.17	12.95	0.88	0.155
LDL-C μmol/g	27.60	26.26	2.15	0.740
HDL-C μmol/g	6.09	6.43	0.66	0.763

TG, triglycerides; TC, total cholesterol; LDL-C, low-density lipoprotein cholesterol; HDL-C, high-density lipoprotein cholesterol. *p* < 0.05 was regarded as statistically significant. *n* = 6 for all groups.

**Table 6:** Effects of high temperature on yolk amino acid of Huaixiang chicken.

Items	NT	HT	SEM	*p*-Value
Total amino acid μmol/g	137.24	117.54	5.20	0.033
Lys mg/g	7.50	8.12	0.32	0.395
Phe mg/g	4.93	4.98	0.09	0.839
Met mg/g	1.74	1.75	0.04	0.961
Thr mg/g	4.89	5.04	0.05	0.181
Ile mg/g	5.19	5.03	0.12	0.565
Leu mg/g	9.75	10.13	0.12	0.810
Val mg/g	5.38	5.40	0.02	0.635
Ala mg/g	4.58	4.36	0.10	0.336
Arg mg/g	6.47	6.94	0.23	0.414
Gly mg/g	3.19	3.37	0.11	0.489
Glu mg/g	5.94	6.02	0.23	0.895
Cys mg/g	1.26	0.52	0.20	0.041
Tyr mg/g	5.98	5.38	0.16	0.035
Pro mg/g	3.94	4.08	0.09	0.506
Ser mg/g	8.00	8.36	0.11	0.101
Asp mg/g	4.55	5.01	0.23	0.367
His mg/g	2.18	2.20	0.03	0.733

*p* < 0.05 was regarded as statistically significant. *n* = 6 for all groups.

## Data Availability

The data presented in this study are available on reasonable request from the corresponding author.
